# Inorganic Characterization of Feeds Based on Processed Animal Protein Feeds

**DOI:** 10.3390/molecules29163845

**Published:** 2024-08-14

**Authors:** Paolo Inaudi, Luca Maria Mercurio, Daniela Marchis, Andrea Bosusco, Mery Malandrino, Ornella Abollino, Laura Favilli, Stefano Bertinetti, Agnese Giacomino

**Affiliations:** 1 Department of Drug Science and Technology, University of Torino, Via Giuria 9, 10125 Torino, Italy; ornella.abollino@unito.it (O.A.); laura.favilli@unito.it (L.F.); 2 Department of Chemistry, University of Torino, Via Giuria 5, 10125 Torino, Italy; luca.m.mercurio@gmail.com (L.M.M.); mery.malandrino@unito.it (M.M.); stefano.bertinetti@unito.it (S.B.); 3 Istituto Zooprofilattico Sperimentale del Piemonte, Liguria e Valle d’Aosta (IZSPLV), 10100 Torino, Italy; daniela.marchis@izsto.it (D.M.); andrea.bosusco@izsto.it (A.B.)

**Keywords:** processed animal proteins, Inorganics, spectroscopy, Ion Chromatography, chemometrics

## Abstract

The potential of utilizing inorganic constituents in processed animal proteins (PAPs) for species identification in animal feeds was investigated, with the aim of using these constituents to ensure the quality and authenticity of the products. This study aimed to quantify the inorganic content across various PAP species and assess whether inorganic analysis could effectively differentiate between PAP species, ultimately aiding in the identification of PAP fractions in animal feeds. Four types of PAPs, namely bovine, swine, poultry, and fish-based, were analyzed and compared to others made up of feathers of vegetal-based feed. Also, three insect-based PAPs (Cricket, Silkworm, Flour Moth) were considered in this study to evaluate the differences in terms of the nutrients present in this type of feed. Ionic chromatography (IC) was used to reveal the concentrations of NO_3_^−^, NO_2_, Cl^−^, and SO_4_^2−^, and inductively coupled plasma optical emission spectroscopy (ICP-OES) to detect Al, Ca, Cd, Cr, Cu, Fe, K, Mg, Mn, Mo, Na, Ni, P, Si, Sr, Ti, and Zn. The application of multivariate chemometric techniques to the experimental results allowed us to determine the identification capability of the inorganic composition to identify correlations among the variables and to reveal similarities and differences among the different species. The results show the possibility of using this component for discriminating between different PAPS; in particular, fish PAPs are high in Cd, Sr, Na, and Mg content; swine PAPs have lower metal content due to high fat; feathers and vegetal feed have similar Al, Si, and Ni, but feathers are higher in Fe and Zn; and insect PATs have nutrient levels comparable to PAPs of other origins but are very high in Zn, Cu, and K.

## 1. Introduction

The enhancements in global quality of life throughout the past century have led to a substantial surge in the demand for raw materials and food resources. Significantly, the consumption of meat has experienced a fourfold increase in the last 60 years, escalating from approximately 80 million tons in 1963 to surpassing 300 million tons in 2019 [1,2]. This surge necessitated the introduction of innovative foods to optimize production yields and accelerate animal rearing processes. As a result, animal by-products have become interesting from both nutritional and economic perspectives. The integration of meat and bone meal (MBM) into animal feed was found to be useful in accelerating the physical growth of animals and mitigating costs associated with processed meat waste. The historical use of abattoir by-products in feed encountered a significant challenge in 1986 with the emergence of bovine spongiform encephalopathy (BSE) among UK cattle [3]. This disease, attributed to the proliferation of infective prions, posed a significant challenge. Following the recognition that the use of MBM as a protein source in feed caused the disease outbreak, emergency measures were implemented, including a complete ban on animal proteins in feeds in the European Union (EU) [4]. This ban was instrumental in containing and gradually reducing the spread of the disease; nonetheless, this prohibition had a major impact on the food industries, as it led to increased costs associated with the treatment of slaughterhouse waste. Prions were discovered by Stanley Prusiner in 1984 while studying another encephalopathy, Scrapie, a disease commonly found in sheep. During the BSE epizootic, Prusiner was able to recognize the role of prions in this new disease [5].

Until the 1980s, when these treatments were discarded because of concerns about the toxicity of the chemicals used, the risks of using MBM remained obscure, as prions in MBM were inactivated by solvents usually used in chemical extractions of fats from abattoir wastes [6]. Subsequent reliance on thermal treatments, ineffective in deactivating prions, resulted in the rapid spread of BSE in the UK and other EU countries.

The spread of BSE lead to the enactment of Regulation (EU) No. 1069/2009 [7], which categorizes animal by-products based on their associated risks: category 1 comprises high-risk products solely intended for incineration; category 2 includes moderate-risk products usable as fertilizer and biogas; category 3 comprises low-risk products. This latter category, following transformation into processed animal proteins (PAP), can be utilized as raw material in the production of feed and pet food (Regulation (EU) 142/2011) [8] and the production of organic fertilizers [7].

In recent years, a significant development in response to this demand has been the emergence of PAP animal-derived products not intended for direct human consumption, such as fats, bones, milk powder, or dried blood [9,10]. Animal by-products, including PAPs, offer elevated levels of fats, proteins, minerals, and essential vitamins, serving as valuable nutritional and energy sources [11]. For example, metals such as iron, manganese, copper, and zinc are recognized as essential trace elements [12]. These trace metals play critical roles in development, growth, and metabolism, participating in various metabolic processes by acting as cofactors of enzymes or providing structural support to proteins. Deficiency or toxicity of these elements can impact human and animal health, giving rise to a number of metabolic and neurological disorders [13,14].

After the implementation of the complete feed ban, various amendments to the legislation were introduced, allowing for the use of specific ingredients such as fishmeal, non-ruminant blood products, collagen and gelatine, hydrolyzed proteins, di- and tri-calcium phosphate of animal origin, as well as egg and dairy products, and farmed insect PAPs in fish feed [15,16].

In a significant development starting from 2021, the European Commission has undertaken a comprehensive review of its prohibitions, granting re-authorization for the inclusion of PAPs derived from pig and poultry in feed intended for poultry, pigs, and fish, avoiding cannibalism, while the use of insect PAPs was extended to pig and poultry feed [17].

Insects represent a valuable protein alternative in food and feed suggested by the Food and Agriculture Organization of the United Nations (FAO) in order to reduce the impact of animal farming [18], as they represent a valuable protein alternative, and their production can result in lower emissions. The legislative framework within the European Union has undergone frequent modifications to effectively regulate the utilization of insects in both food and feed applications. In a comprehensive study conducted by the European Food Safety Authority (EFSA) in 2015, various insect species suitable for feed purposes were identified. The assessment focused on exploring the intricate relationships between production methods, substrates, insect species, and their biological and chemical hazards [14,19].

The EC Insect concept paper of 2016, which provided information to ensure that general feed safety requirements, traceability, and manufacturing requirements apply to insect farming, paved the way for the introduction of insect PAPs in fish feed first, and then in pig and poultry diets [20].

The decision to relax the feed ban aligns with the Farm to Fork strategy [17], which advocates for the enhanced utilization of animal by-product proteins, optimized recycling practices, and the valorization of under-used resources [21].

These initiatives aim to address the EU protein deficit and contribute to the development of a more sustainable food system [22].

Over the years, to meet the demand for the reintroduction of certain PAPs and to comply with European feed bans, various analytical methods have been developed to analyze feeds and their PAP composition. Light microscopy (LM) was declared by the EC in 1998 (Commission Directive EC/88/1998) as the first official method for the detection and characterization of PAPs in feed [23,24]. This method, which was modified and improved several times throughout the years, detects the presence of constituents of animal origin and allows the distinction between terrestrial and fish particles [25]. However, the main limitation of optical microscopy remains the lack of species specificity. Due to changes in legislation, which introduced the need to identify PAP species of origin, in 2013, polymerase chain reaction (PCR) was also accepted as the official method for the detection and characterization of PAP [26]. This method allows reliable species identification but is not able to distinguish the tissue origin of the product. Both the LM and PCR methods showed limitations: microscopy is a complex technique that requires extensive training, is operator-biased, is time-consuming, and does not permit the species identification of animals present in feed; the PCR method is specific and sensitive in nature, but not viable to distinguish between allowed and not allowed ingredients (e.g., milk versus ruminant PAP) [27]. Chromatography coupled to mass spectrometry gave excellent results in identifying and discriminating tiny quantities of constituents subject to regulation, enabling discrimination not only among species but also among tissues of origin [28,29,30].

In recent decades there has been strong interest in defining specific operational protocols and exploring alternative techniques, and many other techniques, such as infrared microscopy, immunoassay methods, and Raman spectroscopy, have just been evaluated as possible alternative methods. All these methods show advantages and limitations [22,28,31,32,33].

Our research proposes the characterization of PAPs from the point of view of the inorganic constituents. The aim of this study was to verify if inorganic analysis could be used to discriminate different PAP species and eventually help discern PAP fractions in animal feeds, in particular, these results could play a crucial role in identifying the origin and authenticity of these products.

## 2. Results and Discussion

### 2.1. ICP-OES Analysis

Table 1 shows the results obtained for the two reference materials using the four considered acid mixtures A, B, C, and D.

The best results were obtained using the D acid mix, which yielded a recovery higher than 70% for all considered elements. For this reason, a mixture of 6 mL HNO_3_ + 1 mL H_2_O_2_ + 2 mL H_2_O + 2 mL HF was chosen for the sample treatment.

Each sample was analyzed in triplicate. A blank, consisting only in the acid solution, undergoing the same microwave procedure, and two external standards prepared in the blank solution were used to obtain a calibration curve.

The concentrations obtained are reported in Table 2. The same results are reported in Tables S1 and S2 in the Supplementary Materials; these tables also report additional information such as the mean, sum, and minimum and maximum values for each element and for each sample. The macroelements registered low variations among animal species, except for Ca and P, due to the importance of skeletal tissue in vertebrates. In all the samples, except for insects, Ca showed the highest concentration, in particular in the poultry, fish, and bovine ones. It is possible to observe the same order of element concentration: Ca > P > Na >/≥ K > Mg. In the insect samples, K and P were the principal components and the order was K > P > Mg > Na > Ca. Vertebrates exhibited higher quantities of P than insects, but P content was still significantly higher in insect feed compared to the 100% vegetal feed, as highlighted by other studies in the literature [34,35,36].

The element concentrations in swine PAPs were generally lower than in other animals. In poultry and fish, we can observe the maximum levels of Ca, P, and Na: the different ratios of fats to total tissues were the possible cause of this trend.

Cadmium concentrations were below the Limit of Detection (LoD_Cd_ = 0.30 mg/kg) in all the samples except fish PAPs. Cd is one of the elements capable of bio-accumulation [37] and is usually found at higher concentrations in marine environments, with natural (erosion of terrains, seasonal washouts) and anthropogenic (industrial activity) origins; in fish species, Cd is accumulated in the kidneys and gills, due to the similarity with Ca^2+^ [38]. Similarly, the high levels of strontium registered in marine fauna can be explained by higher concentrations of Sr in seawater (8 mg/kg versus the 50 µg/kg usually found in fresh water) [39].

The copper and zinc levels were high in all insect species, especially in Acheta Domesticus: insects and shellfish use Cu in hemoglobin-like proteins like hemocyanin; the Zn levels can possibly be explained by its use in structural proteins, as suggested by studies in the literature [40,41].

Iron and silicon were strictly correlated, being crustal elements, and registered high levels in vegetarian animals: while Fe’s importance for mammals is widely known, studies suggest that Si has importance in the development of skeletal tissue in rats and poultry [42].

The manganese and aluminum levels in vegetal feed were high; while Mn is an essential element for plants, Al is a non-essential element. Al-rich soils are usually associated with high Al levels and a reduction in Ca, Mg, and P in leaves [43,44].

Notable high levels of Fe, Mn, Ca were found on feather PAP, a poorly nutritive substrate used mostly as a slow-releasing nitrogen source or hypoallergenic protein source.

### 2.2. Ionic Concentration in Samples: IC

A pilot study was conducted on a single bovine and swine PAP specimen and a single insect (Tenebrio Molitor) for the determination of Cl^−^, NO_2_^−^, NO_3_^−^, and SO_4_^2−^.

The levels of these four ions are shown in Table 3. The same results are reported in Table S3 in the Supplementary Materials; this table also reports additional information such as the mean, sum, and minimum and maximum values for each element and for each sample. NO_2_^−^ was detectable only in fish species; nitrites can achieve higher concentrations in aquatic environments, especially if waters are stagnant. Fish can accumulate nitrites as a consequence of their diet, but also through their gill tissues as a collateral effect of the Cl^−^ uptake cycle.

Chlorides are the most present anion; this is not surprising since chlorides are added as additives to feed.

Nitrates were detected in all the studied species, with high levels in fish, insect, and vegetal feeds; NO_3_^−^ is commonly found in both terrestrial and fish species, with increased concentrations when cooked [45,46]. Insect species’ high levels of NO_3_^−^ and generally high nitrous content relative to their whole weight are key in the nitrous uptake/loss cycle of vegetal species, both directly (carnivorous plants) and indirectly (decomposition of insects’ corpses) [36]. Sulfates are compounds not naturally present in foods, and their excessive concentration can be extremely harmful to human health. Sulfates in foods can derive from pollutants or from permitted technological processes; iron(II) sulfate, for example, is used as a feed additive in animal nutrition [47].

## 3. Chemometric Data Processing

### 3.1. ICP-OES Data

PCA was conducted on the ICP-OES data set to study correlations between the samples. Cumulative variability was described at 95% by the first five PCs, and the correlation matrix observation gave interesting results.

Ca and P had a strong correlation (0.974), a trend observed in other studies; in humans the elements are usually found in a precise ratio of 2.5:1 [48], and for the majority of skeletal tissues, were found in high levels in all the vertebrates species in this study.

Zn and Cu (0.840) were strongly correlated, as were Sr and Cd (0.942): the first two elements registered high concentrations in insects, while the levels of the last two were higher in fish species.

**Figure 1:**
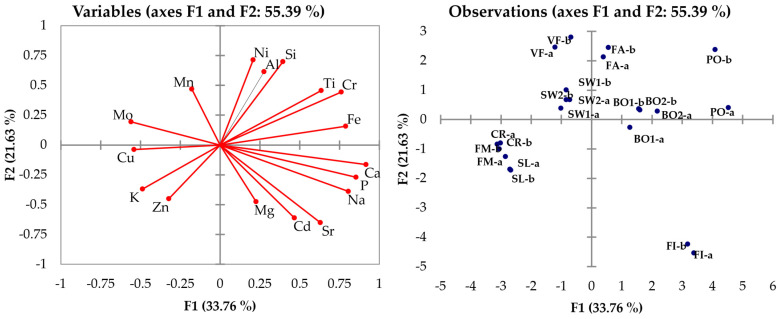
Graph for ICP-OES analysis results.

The variable and observation graphs (Figure 1) highlight the differences between animal species, especially between insects and vertebrates. Swine samples were well separated both from bovine and poultry ones, showing the lowest concentrations of many metals, probably because of the higher ratio of fats in meat. Poultry meat had generally higher concentrations of metals. Fish species were separated from other animals, mainly because of their higher levels of Cd, Na, and Sr. Feather PAP and vegetal feed were surprisingly close, due to their similar content of Si, Al, Ni, and Mn, confirming the principle beyond the use of feather derivatives as fertilizer.

### 3.2. ICP-OES and IC Data

**Figure 2:**
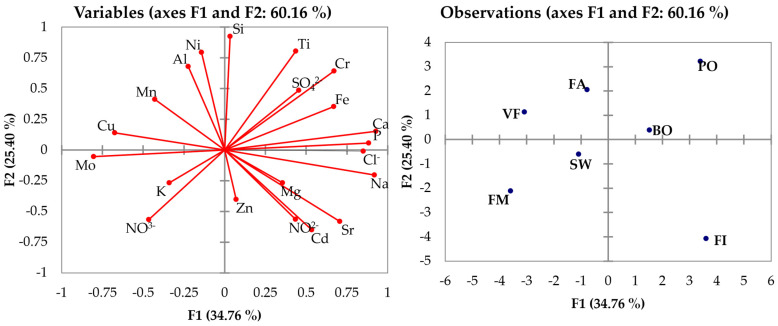
PCA on mean ICP-OES and IC results.

Because of the scarcity of sample quantity, an IC-only chemometric analysis was not viable: the results from the IC analysis were treated with the mean results of ICP-OES, and PCA was conducted on this new set (Figure 2). As in the first PCA, the first five PC described more than 95% of the cumulative variability: the graphs show that Cd, Sr, and NO_2_^−^ were important for ichthyic species, but had little effect in the species’ cluster positions, except for the swine samples.

We can observe a high correlation between Fe and SO_4_^2−^ due to the addition of iron sulfate as a feed additive in animal nutrition, as iron is necessary for the formation of hemoglobin in the blood.

Another correlation is observed between K and NO_3_^−^ due to the use of these salts in feeds; in particular, we can observe that insects present high concentrations of these species, probably because potassium nitrate is widely used as a plant fertilizer.

## 4. Materials and Methods

### 4.1. Samples

Eleven samples (Table 4) were considered for this study: bovine, swine, fish, and poultry’s PAPs, a feather PAP (poultry feathers used on fish farms), and a vegetable feed (pellets and flaked); also, three kinds of insects were considered, namely crickets (Acheta Domesticus), silkworms (Bombix Mori), and moths (Tenebrio Molitors), bred by Kreka (PK Ermelo, Netherlands), a company specialized in insect breeding. The insect samples were received intact, while the feed and the PAPs were already minced [49]. All the samples were provided by Istituto Zooprofilattico Sperimentale del Piemonte Liguria e Valle d’Aosta (IZSPLV).

### 4.2. Sample Pretreatment and Apparatus

The sample pretreatments consisted of microwave-assisted acid mineralization, using polytetrafluoroethylene (PTFE) vessels in a Milestone MLS-1200 Mega (Milestone, Sorisole, Italy) microwave laboratory unit.

To optimize the pretreatment procedure and to assess the accuracy of the results obtained for the determination of the selected analytes, two Standard Reference Materials (SRMs) were used, namely Tomato Leaves 1573a and Bovine Liver Certified Reference Material (CRM) 185. A total of 0.5 g of each SRM was added to the acid mixture. Two different certified materials, similar to the matrix of interest, were chosen to further validate the method.

Four mixtures were tested as extractants in order to find the best acid mix according to our previews works [50]; they are reported in Table 5. Each experiment was performed in triplicate.

The program adopted for acid digestion was as follows: 1—250 W for 1 min; 2—0 W for 1 min; 3—250 W for 5 min; 4—450 W for 5 min; 5—600 W for 5 min.

The solutions obtained were filtered using Watman 5 filters and then diluted to 30 mL with high-purity water (HPW) before the analysis.

The elements (Al, Ca, Cd, Cr, Cu, Fe, K, Mg, Mn, Mo, Na, Ni, P, Si, Sr, Ti, and Zn) were determined using a Perkin Elmer Optima 7000 (Perkin Elmer, Norwalk, CT, USA) ICP-OES.

Standard metal solutions were prepared from concentrated stock solutions (Sigma Aldrich, St. Louis, MO, USA), diluted with HPW produced with a Millipore Milli-Q system (18.2 MΩ cm, Milford, MA, USA) and acid reagents of analytical grade (Sigma Aldrich).

Ionic chromatography was used to reveal the concentrations of NO^3−^, NO^2^, Cl^−^, and SO_4_^2−^. The pretreatments for IC included extraction by agitation in HPW with an Asal VDRL Mod 711+ orbital shaker and 0.45 μm Advantec cellulose filter for vacuum filtration of the obtained solution. Sigma Aldrich standards were used for standard additions, and the solutions were analyzed with a Dionex DX 500 IC with an LC 30 chromatography oven and a an AS9-HC column.

### 4.3. Extraction and Ionic Chromatography

The extraction of ionic species was performed using 15 mL of HPW for 0.5 g of each sample, and a 2 h agitation period, a common method found in the literature [51].

The extract was filtered, diluted by a 1/100 ratio, and analyzed in isocratic conditions with a 9 mM K_2_CO_3_ eluent using the standard addition method to quantify ionic concentrations.

### 4.4. Chemometric Treatment

A chemometric analysis of the results was performed by Principal Component Analysis (PCA) with XLStat 2007.3 software, as a Microsoft Excel add-on.

The multivariate analysis of the data was conducted utilizing the mean values derived from sample replicates.

PCA is a multivariate and unsupervised technique, capable of recognizing specific patterns of correlation and anticorrelation between objects’ variables. It is based on the calculation of principal components (PC), linear combinations of the original values that can be used as new dimensions, thus creating a new space represented by the loading plots, where the operator can find evidence of classes in the data set.

## 5. Conclusions

The concentrations of metals and anions in various PATs highlight their role in providing essential nutrients in animal diets. Chemometric analysis of these components enables the differentiation of PAT origins. Fish PATs are rich in Cd, Sr, Na, and Mg, while swine PATs have lower metal content due to their high fat content. Feathers and vegetal feed contain similar levels of Al, Si, and Ni, but feathers have more Fe and Zn. Insect PATs have nutrient levels comparable to other sources but are very high in Zn, Cu, and K. Microwave digestion followed by ICP-OES analysis aids in assessing pollutant and nutrient content, ensuring quality control, and certifying the animal origin of these products.

## Figures and Tables

**Table 1:** Percentage recoveries (%) obtained during the optimization process.

Tomato Leaves SRM 1537a	A	B	C	D
Al	71.2 ± 6.40	74.7 ± 1.96	63.2 ± 5.94	92.0 ± 2.21
Ba	65.2 ± 1.06	66.5 ± 2.04	63.0 ± 0.32	72.2 ± 1.47
Ca	96.2 ± 2.19	93.9 ± 1.76	93.4 ± 9.00	96.2 ± 1.48
Cd	66.9 ± 0.94	69.8 ± 2.79	67.2 ± 1.82	94.2 ± 1.72
Co	80.8 ± 3.33	77.2 ± 7.24	73.1 ± 1.82	74.0 ± 14.7
Cr	54.0 ± 0.93	50.6 ± 2.72	64.7 ± 1.80	75.8 ± 2.84
Cu	95.3 ± 8.70	72.8 ± 3.15	53.6 ± 8.78	81.4 ± 5.71
Fe	76.3 ± 0.52	78.0 ± 0.87	75.4 ± 1.96	71.7 ± 4.49
K	100 ± 1.75	100 ± 5.62	82.0 ± 10.6	100 ± 3.50
Mg	86.3 ± 1.79	86.4 ± 1.83	85.2 ± 8.72	87.3 ± 4.66
Mn	78.7 ± 1.34	77.2 ± 0.88	78.7 ± 0.92	90.7 ± 0.83
Na	91.6 ± 1.39	88.0 ± 8.19	67.9 ± 1.27	90.6 ± 3.00
P	90.2 ± 1.57	90.1 ± 0.15	83.7 ± 0.42	94.0 ± 0.73
Sr	61.2 ± 1.91	79.6 ± 2.09	76.9 ± 2.35	87.0 ± 0.63
V	59.7 ± 3.71	61.6 ± 0.66	49.8 ± 6.64	72.2 ± 16.2
Zn	77.6 ± 2.86	64.8 ± 0.38	63.4 ± 1.63	78.3 ± 1.53
Mean	78.2	76.9	71.3	84.8
Median	78.1	77.2	70.5	87.1
Bovine Liver CRM 185	A	B	C	D
Ca	91.9 ± 11.7	90.5 ± 2.70	90.4 ± 3.61	92.1 ± 1.92
Cu	79.5 ± 8.2	72.7 ± 0.92	88.1 ± 1.69	88.8 ± 1.34
Fe	86.6 ± 1.12	81.5 ± 1.05	85.0 ± 1.13	87.0 ± 0.71
K	100 ± 1.41	100 ± 7.10	100 ± 3.59	100 ± 11.6
Mg	84.2 ± 1.35	79.2 ± 1.04	71.5 ± 0.88	76.0 ± 0.18
Mn	86.7 ± 11.1	80.9 ± 1.16	77.0 ± 1.74	90.6 ± 0.90
Na	90.8 ± 10.8	84.1 ± 0.98	83.7 ± 1.11	85.2 ± 0.50
P	100 ± 11.2	99.0 ± 1.53	100 ± 4.08	100 ± 9.61
Pb	100 ± 15.3	100 ± 14.6	69.0 ± 14.9	100 ± 12.4
Se	85.1 ± 19.9	88.0 ± 15.6	62.9 ± 26.1	74.7 ± 17.1
Zn	74.5 ± 1.61	72.8 ± 0.39	83.5 ± 0.56	87.9 ± 0.58
Mean	89.0	86.2	82.8	89.3
Median	86.7	84.1	83.7	88.8

**Table 2:** Results for ICP-OES analysis.

	BO1	BO2	SW1	SW2	FI	PO	FA	VF	FM	SL	CR
mg/kg											
Al	84.0 ± 2.5	100 ± 23	4.60 ± 0.35	3.10 ± 0.17	<0.30	49.4 ± 7.7	113 ± 33	118 ± 3	15.9 ± 0.29	0.52 ± 0.07	22.1 ± 0.5
Cd	<0.30	<0.30	<0.30	<0.30	1.20 ± 0.05	<0.30	<0.30	<0.30	<0.30	<0.30	<0.30
Cr	1.33 ± 0.51	1.90 ± 0.32	1.60 ± 0.09	1.89 ± 0.03	1.91 ± 0.09	4.09 ± 1.02	2.21 ± 0.08	1.76 ± 0.24	<0.48	<0.48	0.62 ± 0.19
Cu	6.09 ± 2.54	5.87 ± 2.93	2.71 ± 0.08	4.13 ± 0.37	4.54 ± 3.05	7.71 ± 0.21	11.2 ± 2.8	11.3 ± 0.27	15.7 ± 2.3	8.93 ± 6.70	26.9 ± 1.8
Fe	301 ± 12	288 ± 2	53.8 ± 7.7	53.4 ± 1.4	228 ± 0.89	246 ± 6	279 ± 6	136 ± 1	69.3 ± 2.0	22.3 ± 1.54	84.0 ± 4.9
Mn	8.19 ± 4.69	8.19 ± 4.54	0.73 ± 0.15	0.81 ± 0.87	4.19 ± 0.07	16.6 ± 3.57	23.1 ± 2.15	71.4 ± 18.4	7.96 ± 5.42	10.1 ± 0.15	31.1 ± 4.4
Mo	0.45 ± 0.15	0.59 ± 0.19	0.32 ± 0.62	0.26 ± 0.03	0.15 ± 0.45	0.43 ± 0.65	0.57 ± 2.06	1.20 ± 11.5	1.78 ± 0.2	0.59 ± 0.17	0.74 ± 1.51
Ni	<0.10	<0.10	1.54 ± 0.65	1.15 ± 0.05	<0.10	1.67 ± 0.27	1.15 ± 0.58	1.14 ± 2.48	0.47 ± 0.07	0.31 ± 0.11	0.28 ± 0.11
Si	228 ± 20	248 ± 1	71.0 ± 38.9	119 ± 1	63.7 ± 4.10	320 ± 40	266 ± 15	271 ± 4	133 ± 1	123 ± 4	133 ± 3
Sr	24.7 ± 4.8	27.8 ± 8.0	2.88 ± 0.07	2.63 ± 1.66	228 ± 7	56.6 ± 20.4	13.9 ± 8.5	7.54 ± 7.51	4.12 ± 5.62	1.35 ± 0.4	3.01 ± 0.7
Ti	8.20 ± 0.33	6.22 ± 0.38	0.38 ± 0.91	0.37 ± 27.2	0.22 ± 0.13	32.2 ± 11.0	14.8 ± 3.4	5.49 ± 1.00	1.36 ± 0.17	0.24 ± 0.08	1.76 ± 0.50
Zn	71.5 ± 0.8	74.1 ± 0.28	42.2 ± 0.03	43.9 ± 2.4	131 ± 1	83.6 ± 4.6	120 ± 2	72.2 ± 9.0	129 ± 2.01	125 ± 21	211 ± 18
	g/kg										
Ca	49.1 ± 1.73	57.1 ± 6.10	5.98 ± 0.08	5.76 ± 0.01	73.2 ± 5.98	101 ± 44.6	15.4 ± 2.56	12.0 ± 7.21	0.40 ± 1 × 10^−3^	0.91 ± 0.01	1.35 ± 0.29
K	6.21 ± 0.03	6.17 ± 0.28	0.93 ± 0.01	0.94 ± 0.01	3.24 ± 0.01	3.73 ± 0.40	1.48 ± 0.01	4.51 ± 4.29	10.1 ± 0.02	11.1 ± 0.09	8.75 ± 0.07
Mg	1.49 ± 3 × 10^−3^	1.64 ± 0.11	0.31 ± 4 × 10^−3^	0.31 ± 1 × 10^−3^	2.37 ± 0.15	2.53 ± 0.50	0.74 ± 0.03	1.20 ± 0.64	2.57 ± 0.07	2.70 ± 0.07	0.65 ± 0.03
Na	6.68 ± 0.15	6.91 ± 0.02	0.92 ± 4 × 10^−3^	0.94 ± 0.01	9.06 ± 0.02	7.04 ± 0.14	1.11 ± 0.01	1.20 ± 0.12	1.13 ± 3 × 10^−3^	0.08 ± 4 × 10^−3^	3.60 ± 0.02
P	27.8 ± 1.02	31.7 ± 2.47	4.78 ± 0.02	4.71 ± 0.01	40.9 ± 2.94	57.7 ± 9.20	2.40 ± 0.41	3.71 ± 1.46	8.79 ± 0.04	8.62 ± 0.10	6.50 ± 0.09

**Table 3:** Results (g/kg) for IC analysis.

Anion	BO1	SW1	PO	FI	FA	FM	VF
Cl^−^	3.02	1.78	4.00	3.06	1.26	1.95	0.77
NO_2_^−^	<0.1	<0.1	<0.1	0.12	<0.1	<0.1	<0.1
NO_3_^−^	0.22	0.36	0.30	0.59	0.44	0.64	0.57
SO_4_^2−^	0.30	<0.2	0.38	0.24	0.65	<0.2	<0.2

**Table 4:** Identification code for each sample.

Code	Type of PAP
BO1	Bovine
BO2	Bovine
SW1	Swine
SW2	Swine
FI	Fish
PO	Poultry
FA	Feathers
VF	Vegetal feed
CR	Cricket
SL	Silkworm
FM	Flour moth

**Table 5:** Mixture codes and the corresponding compositions.

Mixture Code	Reagents
A	6 mL HNO_3_ + 1 mL H_2_O_2_
B	6 mL HNO_3_ + 1 mL HCl
C	6 mL HNO_3_ + 1 mL H_2_O_2_ + 1.5 mL H_2_O
D	6 mL HNO_3_ + 1 mL H_2_O_2_ + 2 mL H_2_O + 2 mL HF

## Data Availability

Data is contained within the article or Supplementary Materials.

## Supplementary Materials

The following supporting information can be downloaded at: https://www.mdpi.com/article/10.3390/molecules29163845/s1, Table S1. Results for the ICP-OES analysis for elements at concentration of mg/kg (concentration, mean value, minimum and maximum), Table S2. Results for the ICP-OES analysis for elements at concentration of g/kg (concentration, mean value, minimum and maximum), Table S3. Results (g/kg) for the IC analysis (concentration, mean value, minimum and maximum).
