# Supplementary material for: Inorganic Characterization of Feeds Based on Processed Animal Protein Feeds
Source: Molecules. 2024 Aug 14;29(16):3845. doi: 10.3390/molecules29163845 (PMC11356946; doi:10.3390/molecules29163845)
Supplement: Supplementary file 1 [file molecules-29-03845-s001.zip › molecules-3113676-supplementary.pdf]

Article

Inorganic Characterization of Feeds Based on Processed Animal Proteins Feeds (PAPF)

Paolo Inaudi [1]*, Luca Maria Mercurio [2], Daniela Marchis [3], Andrea Bosusco [3], Mery Malandrino [2], Ornella Abollino [1], Laura Favilli [1], Stefano Bertinetti [2] and Agnese Giacomino [1]*

[1] Department of Drug Science and Technology, University of Torino, Via Giuria 9, Torino, Italy
[2] Department of Chemistry, University of Torino, Via Giuria 5, Torino, Italy
[3] Istituto Zooprofilattico Sperimentale del Piemonte, Liguria e Valle d'Aosta (IZSPLV), 10100 Torino, Italy

Table S1. Results for the ICP-OES analysis for elements at concentration of mg/kg (concentration, mean value, minimum and maximum)

Element	BO1	BO2	SW1	SW2	FI	PO	FA	VF	FM	SL	CR	Mean	Min	Max
Al	84.0 ± 2.5	100 ± 23	4.6 ± 0.35	3.10 ± 0.17	<0.30	49.4 ± 7.7	113 ± 33	118 ± 3	15.9 ± 0.29	0.52 ± 0.07	22.1 ± 0.5	51.06	<0.30(FI)	118(VF)
Cd	<0.30	<0.30	<0.30	<0.30	1.20 ± 0.05	<0.30	<0.30	<0.30	<0.30	<0.30	<0.30	1.20	<0.30	1.20(FI)
Cr	1.33 ± 0.51	1.90 ± 0.32	1.60 ± 0.09	1.89 ± 0.03	1.91 ± 0.09	4.09 ± 1.02	2.21 ± 0.08	1.76 ± 0.24	<0.48	<0.48	0.62±0.19	1.92	<0.48(FM,SI)	4.09(PO)
Cu	6.09 ± 2.54	5.87 ± 2.93	2.71 ± 0.08	4.13 ± 0.37	4.54 ± 3.05	7.71 ± 0.21	11.2 ± 2.8	11.3 ± 0.27	15.7 ± 2.3	8.93 ± 6.70	26.9 ± 1.8	9.55	2.71(SW1)	26.9(CR)
Fe	301 ± 12	288 ± 2	53.8 ± 7.7	53.4 ± 1.4	228 ± 0.89	246 ± 6	279 ± 6	136 ± 1	69.3 ± 2.0	22.3 ± 1.54	84.0 ± 4.9	160	22.3(SL)	301(BO1)
Mn	8.19 ± 4.69	8.19 ± 4.54	0.73 ± 0.15	0.81 ± 0.87	4.19 ± 0.07	16.6 ± 3.57	23.1 ± 2.15	71.4 ± 18.4	7.96 ± 5.42	10.1 ± 0.15	31.1 ± 4.4	16.6	0.73(SW1)	71.4(VF)
Mo	0.45 ± 0.15	0.59 ± 0.19	0.32 ± 0.62	0.26 ± 0.03	0.15 ± 0.45	0.43 ± 0.65	0.57 ± 2.06	1.20 ± 11.5	1.78 ± 0.2	0.59 ± 0.17	0.74 ± 1.51	0.64	0.15(FI)	1.78(FM)
Ni	<0.10	<0.10	1.54 ± 0.65	1.15 ± 0.05	<0.10	1.67 ± 0.27	1.15 ± 0.58	1.14 ± 2.48	0.47 ± 0.07	0.31 ± 0.11	0.28 ± 0.11	0.96	<0.10(BO,FI)	1.67(PO)
Si	228 ± 20	248 ± 1	71.0 ± 38.9	119 ± 1	63.7 ± 4.10	320 ± 40	266 ± 15	271 ± 4	133 ± 1	123 ± 4	133 ± 3	180	63.7(FI)	320(PO)
Sr	24.7 ± 4.8	27.8 ± 8.0	2.88 ± 0.07	2.63 ± 1.66	228 ± 7	56.6 ± 20.4	13.9 ± 8.5	7.54 ± 7.51	4.12 ± 5.62	1.35 ± 0.4	3.01 ± 0.7	33.9	1.35(SL)	228(FI)
Ti	8.20 ± 0.33	6.22 ± 0.38	0.38 ± 0.91	0.37 ± 27.2	0.22 ± 0.13	32.2 ± 11.0	14.8 ± 3.4	5.49 ± 1.00	1.36 ± 0.17	0.24 ± 0.08	1.76 ± 0.50	6.48	0.22(FI)	32.2(PO)
Zn	71.5 ± 0.8	74.1 ± 0.28	42.2 ± 0.03	43.9 ± 2.4	131 ± 1	83.6 ± 4.6	120 ± 2	72.2 ± 9.0	129 ± 2.01	125 ± 21	211 ± 18	100	42.2(SW1)	211(CR)
Sum	733	761	182	231	663	818	845	697	379	292	515			
Max	301(Fe)	288(Fe)	71.0(Si)	119(Si)	228(Fe)	320(Si)	279(Fe)	271(Si)	133(Si)	125(Zn)	211(Zn)			

Table S2. Results for the ICP-OES analysis for elements at concentration of g/kg (concentration, mean value, minimum and maximum)

Element	BO1	BO2	SW1	SW2	FI	PO	FA	VF	FM	SL	CR	Mean	Min	Max
Ca	49.1	57.1 ± 6.10	5.98 ± 0.08	5.76 ± 0.01	73.2 ± 5.98	101 ± 44.6	15.4 ± 2.56	12.0 ± 7.21	0.40 ± $1*10^{-3}$	0.91 ± 0.01	1.35 ± 0.29	29.3	0.40(FM)	101(PO)
K	6.21 ± 0.03	6.17 ± 0.28	0.93 ± 0.01	0.94 ± 0.01	3.24 ± 0.01	3.73 ± 0.40	1.48 ± 0.01	4.51 ± 4.29	10.1 ± 0.02	11.1 ± 0.09	8.75 ± 0.07	5.20	0.93(SW)	11.1(SL)
Mg	1.49 ± $3*10^{-3}$	1.64 ± 0.11	0.31 ± $4*10^{-3}$	0.31 ± $1*10^{-3}$	2.37 ± 0.15	2.53 ± 0.50	0.74 ± 0.03	1.20 ± 0.64	2.57 ± 0.07	2.70 ± 0.07	0.65 ± 0.03	1.50	0.31(SW)	2.70(SL)
Na	6.68 ± 0.15	6.91 ± 0.02	0.92 ± $4*10^{-3}$	0.94 ± 0.01	9.06 ± 0.02	7.04 ± 0.14	1.11 ± 0.01	1.20 ± 0.12	1.13 ± $3*10^{-3}$	0.08 ± $4*10^{-3}$	3.60 ± 0.02	3.52	0.08(SL)	9.06(FI)
P	27.8 ± 1.02	31.7 ± 2.47	4.78 ± 0.02	4.71 ± 0.01	40.9 ± 2.94	57.7 ± 9.20	2.40 ± 0.41	3.71± 1.46	8.79 ± 0.04	8.62 ± 0.10	6.50 ± 0.09	18.0	2.40(FA)	57.7(PO)
Sum	91.3	104	12.9	12.7	128	172	21.1	22.6	23.0	23.4	20.8			
Min	1.49(Mg)	1.64(Mg)	0.31(Mg)	0.31(Mg)	2.37(Mg)	2.53(Mg)	0.74(Mg)	1.20(Mg)	0.40(Ca)	0.08(Na)	0.65(Mg)			
Max	49.1(Ca)	57.1(Ca)	5.98(Ca)	5.76(Ca)	73.2(Ca)	101(Ca)	15.4(Ca)	12.0(Ca)	8.79(P)	11.1(K)	8.75(K)			

Table S3. Results (g/kg) for the IC analysis (concentration, mean value, minimum and maximum)

Anion	BO1	SW1	PO	FI	FA	FM	VF	Mean	Min	Max
Cl^-	3.02	1.78	4.00	3.06	1.26	1.95	0.77	2.26	0.77(VF)	4.00(PO)
NO_2^-	< 0.1	< 0.1	< 0.1	0.12	< 0.1	< 0.1	< 0.1	-	<0.1	0.12(FI)
NO_3^-	0.22	0.36	0.30	0.59	0.44	0.64	0.57	0.45	0.22(BO1)	0.64(FM)
SO_4^{2-}	0.30	< 0.2	0.38	0.24	0.65	< 0.2	< 0.2	0.39	<0.2(SW,FM,VF)	0.65(FA)
Sum	3.54	2.14	4.68	4.01	2.35	2.59	1.34			
Min	<0.1(NO_2^-)	<0.1(NO_2^-)	<0.1(NO_2^-)	0.12(NO_2^-)	<0.1(NO_2^-)	<0.1(NO_2^-)	<0.1(NO_2^-)			
Max	3.02(Cl-)	1.78(Cl-)	4.00	3.06(Cl-)	1.26(Cl-)	1.95(Cl-)	0.77(Cl-)			
